# Time to Rethink Refugee and Migrant Health in Europe: Moving from Emergency Response to Integrated and Individualized Health Care Provision for Migrants and Refugees

**DOI:** 10.3390/ijerph15061100

**Published:** 2018-05-28

**Authors:** Karl Puchner, Evika Karamagioli, Anastasia Pikouli, Costas Tsiamis, Athanasios Kalogeropoulos, Eleni Kakalou, Elena Pavlidou, Emmanouil Pikoulis

**Affiliations:** International Medicine—Health Crisis Management, Medical School, NKUA, Dilou1 & M. Asias, 11527 Athens, Greece; karl.puchner@gmx.de (K.P.); pikoulianastasia@gmail.com (A.P.); ctsiamis@med.uoa.gr (C.T.); kardamyla.chios@gmail.com (A.K.); ekakalou@yahoo.gr (E.K.); lenapvld@gmail.com (E.P.); mpikoul@med.uoa.gr (E.P.)

**Keywords:** refugee and migrant (R&M) health, refugee crisis, healthcare, European Union (EU)

## Abstract

In the last three years, the European Union (EU) is being confronted with the most significant influx of migrants and refugees since World War II. Although the dimensions of this influx—taking the global scale into account—might be regarded as modest, the institutional response to that phenomenon so far has been suboptimal, including the health sector. While inherent challenges of refugee and migrant (R&M) health are well established, it seems that the EU health response oversees, to a large extend, these aspects. A whole range of emergency-driven health measures have been implemented throughout Europe, yet they are failing to address adequately the changing health needs and specific vulnerabilities of the target population. With the gradual containment of the migratory and refugee waves, three years after the outbreak of the so-called ‘refugee crisis’, we are, more than ever, in need of a sustainable and comprehensive health approach that is aimed at the integration of all of migrants and refugees—that is, both the new and old population groups that are already residing in Europe—in the respective national health systems.

## 1. Introduction

According to the World Health Organization (WHO), both at the global and national levels, the health policies and strategies to manage the health consequences of migration and displacement have failed to keep up with the speed and diversity of modern migration and displacement [1]. The approaches are frequently fragmented and costly, sometimes operating in parallel to national health systems, and may depend on external funding, which can lack sustainability. In addition to the systemic response deficiencies that have been observed, refugee and migrant (R&M) health as a field, exhibits significant inherent challenges as it is dealing with people that are in transit, with multiple vulnerabilities within an unstable sociopolitical context. Although often underestimated, the exposure to health risks during the transit phase is immense. Evidence from past refugee and migrant movements show that, depending on the context, up to more than 20% of the population on the move might die on the grounds of murder, illness, or accidents [2]. Apart from the disproportionally high mortality rate, R&M populations exhibit a series of specific vulnerabilities that pose further risks for their health status. Prolonged fear, chronic anxiety, low self-esteem, loss of control, and alienation are common emotional states among R&M—it is known that chronic exposure to these may have a detrimental effect on health [3]. However, the increased vulnerability of R&M is not only caused by adverse emotional states but also by the underlying structural factors influencing the basic social determinants of health. The major impact that social determinants may have on health status is widely recognized, with the WHO proclaiming action on social justice as a top health policy priority [4]. In particular, poor or insecure housing, higher exposure to and lesser protection from violence, barriers to employment, the legal, and the educational and the health system are potent morbidogenic conditions which R&Ms have often to endure in host countries [5,6]. Finally, it should not be forgotten that R&M movements occur in and/or trigger an unstable sociopolitical context and they are frequently also characterized by changing epidemiological patterns, depending on the transitional phase. Thus, while acute and pressing health problems, such as accidents and acute infections, typically dominate the epidemiology of first phase of transition, it is with provisional or permanent settlement that the full burden of chronic diseases and mental health illness unfolds [7].

This manuscript aims to assess the current R&M health response and discuss the necessity for an integrated and individualized health care provision model for migrants and refugees in the European context.

## 2. R&M Health in the European Context

For the first time after World War II, the country members of the European Union (EU) have been confronted with a significant influx of R&M. Contrary to the prior mass population movements that were unfolding in Europe, the current influx consists of a highly inhomogeneous population of third country nationals with diverse ethnical and social backgrounds, migration motives, and legal status, and on arrival, these people may have and/or can achieve in the respective host country based on international, national, and EU law (i.e., asylum seekers, refugee, migrants, and undocumented migrants). During the peak year of the R&M movements, in 2015, the UNHCR registered 1,015,078 sea arrivals on EU territory. However, within the context of the EU–Turkey deal and other bilateral agreements with neighboring states, which are aimed at containment of the R&M movements towards Europe, the arrivals had dropped to less than 171,000 in 2017 [8]. With more than 65 million forcibly displaced people living worldwide and a global migrant population of 258 million people [9], it becomes evident that the recent R&M wave that is reaching the EU is of a rather moderate scale. Given the substantial demographic and huge economic size of the EU—its share of the world GDP reaches 23%—it is highly unlikely that the recent R&M wave overstretches the immigration capacity of the member states [10]. Yet, surprisingly enough, the term ‘refugee crisis’, which emerged in 2015, has dominated ever since the public discourse and has substantially influenced the political landscape both at a European and national level. In line with this rather abrupt reaction of the public opinion, most of the policy response to the R&M wave has been emergency driven without any long-term scope, common vision, and/or acting among the EU-member states, which often results in desultory, uncoordinated, or even contradictory responses at all levels, including the field of R&M health.

A multitude of stakeholders, from (non-governmental organizations) NGOs to ministries and European authorities with various mandates and portfolios, have co-acted in financing and providing provisional health services to R&M during the past few years. Yet, the success of this mass mobilization of resources and manpower, in terms of health outcomes, is rather modest. With the extremely limited legal pathways that are offered to people seeking international protection by the EU, the unsafe trespassing of the Mediterranean Sea, was and remains, even three years after the peak of the recent R&M wave, the predominant entry route into Europe. The dangerous voyage, in combination with the indecisiveness of the EU and its member states to establish an effective sea rescue response mechanism, poses a life-threatening situation—it is estimated that more than 12,000 people have died or disappeared along the trespassing routes in the Mediterranean Sea since 2015 [8]. It should also be added that the lack of legal pathways and safe passage for people on the move favors a dependency on smuggler and trafficking networks, which automatically exacerbates their exposure to violence and insecurity [11]. Although, as in every R&M mass movement, the transit accommodation in camps and other types of mass accommodation often constitutes an inevitable interim solution, and little has been done in order to prevent health hazards that arise from overcrowding and prolonged stay under poor living conditions. Numerous reports on measles and varicella outbreaks in refugee camps throughout the EU are indicative of a deferred and/or delayed vaccination coverage of this vulnerable population, while the frequently observed scabies and sporadic Hepatitis A outbreaks are suggestive of the negligence of basic water, sanitation, and hygiene measures by the respective public health authorities [12,13,14]. These, by all means preventable, outbreaks sustain the common but ill-founded concern of R&M spreading infectious diseases. Prolonged or consecutive stays in camps and reception centers, were and still are routine practice in many member states, substantially aggravating the health status, particularly of the most vulnerable, that is, of the children, multimorbid patients, and people living with disabilities [15]. In addition, it becomes evident that the health authorities throughout the EU have grossly underestimated the importance of mental health services with protracted and grave psychiatric manifestations being currently a frequent phenomenon among the population that are under discussion [16,17]. Tailored interventions targeting systematically highly prevalent health problems among the R&M populations, such as exposure to sexual and gender based violence (SGBV) and post traumatic stress disorders (PTSD), are still rarely encountered in the EU context [18]. There is enough evidence showing that restricting access to health care for R&M is not only unfavorable for the health status of this population but it is also economically counterproductive for the health system of the host country [19]. Contrary to that evidence, the health policy for R&M in the EU remains predominantly restrictive. In particular, unconditional and free access to healthcare for undocumented migrants is restricted to emergency services in most of the EU member states, leading to a de facto exclusion of this population group from the majority of preventive healthcare services [20]. Furthermore, legislation reform for equalizing access to the health care of refugees/asylum seekers with the rest of the insured population is still pending in many countries, such as Germany, Denmark, and Belgium [21]. In this context, it is also worth mentioning that only 13 of the 28-member states offer free of charge interpreting services to patients, while none of the EU member states have, until now, an official health strategy targeting migrants [21,22].

## 3. The Need to Move from Emergency Response to Integrated and Individualized Health Care Provision for Migrants and Refugees

Three years after the peak influx of R&M in 2015 into the EU territory, the EU health response is still predominantly emergency-driven. Although the new arrivals are gradually contained, the policies and operations, including health, remain short-term financed, short-sighted, and essentially unharmonized with the international obligations of the EU. One of the most blatant examples of these failures is the current border management, which appeals to a prolonged ‘emergency state’, and primarily consists of policing, securitization, and off-shoring and out-sourcing of activities that are aimed at fending off immigrants. Thus, it is inherently in conflict with the human rights framework and the Health in All Policies (HiAP) approach, to which the EU is officially committed. Furthermore, it should be noted that the EU has recently made explicit commitments under the UN Sustainable Development Goals, which recognizes the positive contribution of refugees and migrants to have inclusive growth and sustainable development, and to promote universal health coverage of all people, irrespective of their legal status [23]. Notwithstanding the encouragement of developments at the policy level, such as the recent recast of the Reception Directive, which contains improvement and harmonization of the reception conditions throughout the EU [24], a further improvement of legislation, harmonization, and broadening of entitlement to free health care is urgently needed for both the new R&M and the majority of R&M that are already residing in Europe. Parallel to this, intensified efforts that are aimed at assessing and tackling huge public health problems of the R&M population, such as mental health impairment, vaccination coverage, and SGBV, are essential. These measures, although requiring substantial investments, can pave the path to full integration of the majority of the R&Ms in the respective country health system, significantly improving the health status of the general population and significantly reducing health expenditure in the long run.

**Figure 1:**
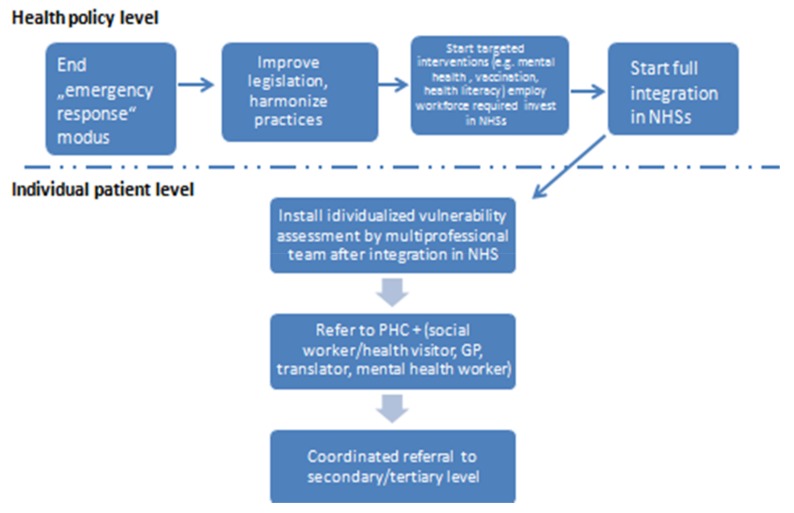
Integrated and individualized health care healthcare provision scheme.

In addition to the actions at the policy level, a comprehensive and tailored operational approach is needed in order to ensure a smooth integration of the individual patients into the respective health systems. A multidisciplinary assessment of social determinants, such as the housing situation, legal, employment, and insurance status, at the very first contact with the health system is key in order to start addressing the parallel health needs and pressing social issues that can significantly affect the health status of the individual. A timely linkage, after first contact with the health system to a primary health care unit with extended health, social, and interpretation services, will thus allow the prioritization of problems and coordinated referral, and could further enhance the integration of the individual patient in the respective health system, without overstretching the capacities of the secondary and tertiary health care level (Figure 1).

## 4. Conclusions

In the face of the current situation, it is key to not only to deal with the short-term needs of the recently arrived R&M, but to also to integrate long-term R&M health responses in the respective health systems of the member states and thus strengthen the public health at the national and European level. Access to responsive, people-centered health systems is essential in order to ensure good quality health care for all refugees, asylum seekers, and migrants, not only during their migration journey but also after settlement in their respective host countries. This implies mainstreaming of R&M health in all European and national policies that are related to migration; reducing R&M specific vulnerabilities; overcoming formal and informal barriers to health care, such as language, administrative hurdles, or lack of information about health entitlements and meeting the needs of all of the R&Ms, irrespective of their legal status.
